# Inhibitory single neuron control of seizures and epileptic traveling waves in humans

**DOI:** 10.1186/1471-2202-15-S1-F3

**Published:** 2014-07-21

**Authors:** Omar J Ahmed, Mark A Kramer, Wilson Truccolo, Jason S Naftulin, Nicholas S Potter, Emad N Eskandar, Garth R Cosgrove, Andrew S Blum, Leigh R Hochberg, Sydney S Cash

**Affiliations:** 1 Department of Neurology, Harvard Medical School and Massachusetts General Hospital, Boston, MA 02114, USA; 2 Department of Neurosurgery, Harvard Medical School and Massachusetts General Hospital, Boston, MA 02114, USA; 3 Department of Neurosurgery, Rhode Island Hospital, Providence, RI 02903, USA; 4 Department of Neurology, Rhode Island Hospital, Providence, RI 02903, USA; 5 Department of Neuroscience, Brown University, Providence, RI 02912, USA; 6 School of Engineering, Brown University, Providence, RI 02912, USA; 7 Department of Mathematics & Statistics, Boston University, MA 02215, USA

## 

Inhibitory neuronal activity is critical for the normal functioning of the brain, but is thought to go awry during neurological disorders such as epilepsy. Animal models have suggested both decreased and increased inhibition as possible initiators of epileptic activity, but it is not known if, or how, human inhibitory neurons shape seizures. Here, using large-scale recordings of neocortical single neurons in patients with secondarily generalized tonic-clonic seizures, we show that fast-spiking (FS) inhibitory activity first increases as a seizure spreads across the neocortex, impeding and altering the spatial flow of fast epileptic traveling waves. Unexpectedly, however, FS cells cease firing less than half-way through a seizure. We use biophysically-realistic computational models to show that this cessation is due to FS cells entering depolarization block as a result of extracellular potassium accumulation during the seizure and not because they are inhibited by other inhibitory subtypes. Strikingly, this absence of FS inhibitory activity is accompanied by dramatic increases in local seizure amplitude along with unobstructed traveling waves and is seen during all secondarily generalized seizures examined, independent of etiology or focus. FS cessation also leads to prominent spike-and-wave events, suggesting that FS cell dynamics control the transition between the tonic and clonic phases of these seizures. Thus, it may be possible to curtail human seizures by preventing inhibitory neurons from entering potassium-dependent depolarization block, a novel and potentially powerful therapeutic avenue in treating multiple kinds of epilepsies.

